# Enhancing the efficacy of cisplatin in ovarian cancer treatment – could arsenic have a role

**DOI:** 10.1186/1757-2215-2-2

**Published:** 2009-01-14

**Authors:** C William Helm, J Christopher States

**Affiliations:** 1 Department of Obstetrics, Gynecology & Women's Health, University of Louisville School of Medicine, Louisville KY 40292, USA; 2 Department of Pharmacology & Toxicology, University of Louisville School of Medicine, Louisville KY 40292, USA

## Abstract

Ovarian cancer affects more than 200,000 women each year around the world. Most women are not diagnosed until the disease has already metastasized from the ovaries with a resultant poor prognosis. Ovarian cancer is associated with an overall 5 year survival of little more than 50%. The mainstay of front-line therapy is cytoreductive surgery followed by chemotherapy. Traditionally, this has been by the intravenous route only but there is more interest in the delivery of intraperitoneal chemotherapy utilizing the pharmaco-therapeutic advantage of the peritoneal barrier. Despite three large, randomized clinical trials comparing intravenous with intraperitoneal chemotherapy showing improved outcomes for those receiving at least part of their chemotherapy by the intraperitoneal route.

Cisplatin has been the most active drug for the treatment of ovarian cancer for the last 4 decades and the prognosis for women with ovarian cancer can be defined by the tumor response to cisplatin. Those whose tumors are innately platinum-resistant at the time of initial treatment have a very poor prognosis. Although the majority of patients with ovarian cancer respond to front-line platinum combination chemotherapy the majority will develop disease that becomes resistant to cisplatin and will ultimately succumb to the disease.

Improving the efficacy of cisplatin could have a major impact in the fight against this disease. Arsenite is an exciting agent that not only has inherent single-agent tumoricidal activity against ovarian cancer cell lines but also multiple biochemical interactions that may enhance the cytotoxicity of cisplatin including inhibition of deoxyribose nucleic acid (DNA) repair. In vitro studies suggest that arsenite may enhance the activity of cisplatin in other cell types. Arsenic trioxide is already used clinically to treat acute promyelocytic leukemia demonstrating its safety profile. Further research in ovarian cancer is warranted to define its possible role in this disease.

## Review

Epithelial ovarian cancer (EOC) affects approximately 204,000 women a year worldwide and is responsible for about 125,000 deaths [1]. The American Cancer Society estimates that in the USA alone the disease will be diagnosed in 21,650 women and cause the death of 15,520 women during 2008 [2]. It is often called the 'silent killer' because it causes few symptoms until it has metastasized within the peritoneal cavity at which time the chance of cure is markedly reduced. Although great strides have been made in the treatment of EOC, the enigma remains that a disease which is highly sensitive to chemotherapy compared to many other types of cancer is associated with an overall 5 year survival of just over 50% [3-6].

### Cytoreductive Surgery

The management of advanced EOC has evolved over the last 30 years to become a combination of initial cytoreductive surgery (CRS) followed by chemotherapy. In 1968 Munnell reported an improved survival in patients who had maximal CRS compared to partial removal or biopsy only [7] and over the years, many retrospective reports have confirmed this finding [8-11]. Although no randomized studies have been performed the role of surgery was supported in a meta-analysis of 6885 patients undergoing CRS during the 'platinum era' where on an institutional basis for each 10% increase in the percentage of patients undergoing maximal CRS there was a 5.5% increase in median survival duration [12].

The reason CRS is thought to be effective when combined with chemotherapy is that it removes bulky disease containing poorly-oxygenated, non-proliferating cells which are either resistant to chemotherapy now, or potentially could become resistant, and leaves small volume tumors with a higher proportion of cells in the proliferative phase making them more susceptible to chemotherapy. At one time the concept of 'optimal' residual disease at completion of initial CRS for EOC was accepted as being any nodule < 2 cm in dimension [13] but it is now established that the most favorable prognosis is in patients with no macroscopic residual disease at all [14]. Unfortunately, 'no macroscopic disease' does not signify the complete absence of disease because so many patients in this situation at the end of surgery experience recurrence following front-line treatment. No less than 60% of patients who present with advanced disease and have a complete pathologic response to front-line therapy documented at second-look surgery will recur [15].

### Chemotherapy

The most active chemotherapy agents in ovarian cancer are the platinum analogues, cisplatin and carboplatin. The antitumor activity of cisplatin (cis-diamminedichloroplatinum (II)) was discovered by Rosenberg and colleagues in 1961 [16]. Initial studies demonstrated that the whilst the agent had significant activity against several tumor types patients experienced severe renal and gastrointestinal toxicity [17]. Later it was shown that renal toxicity could be minimized by aggressive prehydration and diuresis [18,19]. Cisplatin was introduced in the late 1970's and platinum-based combination chemotherapy became the most frequently used treatment for EOC. In a trial of single agent therapy, cisplatin was shown to be better than a previously favored agent cyclophosphamide [20]. Three major trials established cisplatin combination therapy as the standard regimen in advanced EOC [21]. A study randomizing patients with advanced EOC to cyclophosphamide with or without cisplatin reported better outcomes in the combination arm [22]. A Gynecologic Oncology Group study which included over 200 patients with advanced EOC reported that patients randomized to treatment with doxorubicin and cyclophosphamide with or without cisplatin had significantly better responses in the cisplatin containing arm [23]. A Dutch study reported a better outcome for a cisplatin containing regimen over combination hexamethylmelamine, cyclophosphamide, methotrexate, 5-fluorouracil (HexaCAF) [24]. The evidence was further supported in a meta-analysis of 45 trials including over 8000 patients with EOC treated with or without cisplatin. Survival was better with platinum alone and with platinum-containing combinations [25].

An additional class of drug, the taxanes, was discovered and came to play a role in the front-line armamentarium against EOC. In 1971 paclitaxel was identified as the active constituent of an extract of the bark of the Pacific yew tree, *Taxus brevifolia *[26,27]. In early clinical trials on recurrent EOC paclitaxel was associated with an overall response rate of 36% [28]. It became established as the combination agent of choice with cisplatin after a Gynecologic Oncology Group study in women with advanced, suboptimally cytoreduced EOC showed a significantly better median overall survival in patients randomized to receive intravenous (IV) paclitaxel/cisplatin (37.5 months) in comparison with cyclophosphamide/cisplatin (24.4 months) [3]. Paclitaxel and subsequently its cousin, docetaxel were shown to have a unique mechanism of action binding to tubulin polymers (microtubules) and stabilizing the microtubule against depolymerization [29-32].

During this time analogues of cisplatin were investigated in an effort to maintain efficacy with reduced toxicity. Carboplatin was developed by substituting a cyclobutanedicarboxylate moiety for the two chloride ligands of cisplatin. Phase I and II trials of carboplatin showed that it was much less toxic than cisplatin especially with regard to neurotoxicity, nephrotoxicity and emetogenicity whilst retaining significant chemotherapeutic activity [33-37]. Many trials have been performed comparing cisplatin and carboplatin alone or in combination in patients with EOC and two meta-analyses found no difference in survival [25,38]. A large, randomized trial comparing intravenous docetaxel with either cisplatin or carboplatin showed equivalency [39] and following initial front-line CRS, intravenous administration of cisplatin or carboplatin together with a taxane, either paclitaxel or docetaxel, has become the standard therapy for patients with EOC [3,39].

### Intraperitoneal Chemotherapy

For over twenty years there has been interest in the delivery of intraperitoneal therapy for ovarian cancer in order to maximize the efficacy and reduce the toxicity. Dedrick proposed that the intraperitoneal delivery of certain chemotherapeutic agents could lead to a significant increase in peritoneal cavity drug exposure compared to that in the systemic vascular compartment [40]. For drugs most active in EOC the ratio of their intraperitoneal to plasma concentrations varies from 18–20× for carboplatin and cisplatin to 120 – > 1000× for the taxanes, docetaxel and paclitaxel [41]. EOC should be a good target for intraperitoneal treatment because it is relatively chemo-sensitive and the cancer remains confined within the peritoneal cavity for much of its natural history. Three large randomized studies [42-44] have each shown improved responses for intraperitoneal (IP) delivery and a meta-analysis of all studies reported a clear benefit for patients receiving at least part of their front-line therapy by the IP route [45]. A recent study (Gynecologic Oncology Group protocol #172) examining experimental intravenous/intraperitoneal (IV/IP) chemotherapy for EOC showed a significant increase in overall survival in those receiving IP chemotherapy from 49 months to 66 months [44]. The National Cancer Institute has suggested that IP chemotherapy should be offered for patients' consideration for front-line treatment of ovarian cancer [46]. Despite large randomized trials indicating benefit, the use of intraperitoneal therapy in EOC is neither offered to the majority of eligible women nor accepted as standard of care by many oncologists

Despite the advances in the treatment of EOC much more effective therapy is necessary. This is exemplified by the results of Gynecologic Oncology Group protocol #172 where even in the IP/IV arm which led to median extension of survival of 16 months over patients treated only with IV therapy the recurrence rate was 65% within the period of the study. This recurrence rate is the current optimum situation in EOC.

One way of improving outcome for patients with EOC is to develop novel methods of enhancing the activity of cisplatin. Ovarian cancers that are resistant to platinum therapy are either innately resistant, shown by a lack of response to front-line therapy, or develop platinum resistance during the cancer's life history. In the patient this is demonstrated by an initial response to platinum agents followed by development of platinum resistance as the cancer progresses. Most of the women die with platinum resistant disease. Methods of preventing or overcoming resistance to cisplatin thus could be extremely beneficial.

### Cisplatin Resistance

Cisplatin reacts preferentially with the N7 position guanine to form a variety of monofunctional and bifunctional adducts which result in intrastrand or interstrand cross-links, effectively preventing normal DNA function [17,47]. Platinum resistance mechanisms fall into two main groups: A) those that limit the formation of cytotoxic platinum-DNA adducts and B) those that prevent cell death from occurring after platinum-DNA adduct formation. Group A includes decreased drug accumulation and increased drug inactivation by cellular protein and non-protein thiols whilst Group B includes increased platinum-DNA adduct repair and increased platinum-DNA damage tolerance [17].

Cisplatin accumulates within the cell by passive diffusion or facilitated transport [48] and the majority of cell lines that have been selected for cisplatin resistance in vitro show a decreased platinum accumulation phenotype most likely due to decreased uptake rather than enhanced drug efflux[17]. There are few experimental methods currently known to increase platinum uptake into cells but one method is to deliver it with mild hyperthermia. Hyperthermia has been shown to increase the cytotoxicity of cisplatin and other chemotherapeutic agents in both human cell culture and animal models [49-53]. Investigations of the cellular effects of the combination have demonstrated increased DNA cross-linking and increased DNA adduct formation [50,54]. It has also been shown that cisplatin penetrates deeper into peritoneal tumor implants when delivered intraperitoneally with hyperthermia [54]. The mechanism of the effect of hyperthermia on cisplatin cytotoxicity and the role it might play in treatment awaits further investigation.

Multidrug resistance protein (MRP) is a member of a family of transport proteins that facilitates the extrusion of a variety of glutathione-coupled and unmodified drugs out of cells but over-expression of MRP alone does not confer resistance [55]. With regard to inactivation of platinum, the formation of conjugates between glutathione (GSH) and platinum drugs may be an important step for their inactivation and elimination from the cell [17]. There is a strong association between increased platinum drug sensitivity and lower GSH levels [56]. However, reducing GSH levels with drugs such as buthionine sulfoximine has resulted in only low to modest potentiation of cisplatin sensitivity [57]. It has been suggested that this may be due to the fact that formation of GSH-platinum conjugates is a slow process [58].

Inactivation may also occur by binding of the platinum drugs to metallothionein (MT) proteins. MTs are a family of sulfhydryl-rich, small molecular weight proteins that participate in heavy metal binding and detoxication. Modulation of MT levels can alter cisplatin sensitivity but the contribution of MT to clinical platinum drug resistance is unclear [17]. In some cell lines, elevated MT levels have been shown to be associated with cisplatin resistance, whereas in others, they have not [59,60].

Once platinum-DNA adducts are formed, cells must either repair or tolerate the damage in order to survive. The capacity to repair DNA damage rapidly and efficiently plays a role in determining a tumor cell's sensitivity to platinum drugs [17]. Increased repair of platinum-DNA lesions in cisplatin-resistant cell lines has been compared with their sensitive counterparts in several human cancer cell lines, including ovarian cancer [61,62]. Repair of platinum-DNA adducts occurs predominantly by nucleotide excision repair (NER) [17].

Inhibiting DNA repair activity to enhance platinum drug sensitivity has been an active area of investigation. Agents that have been used include nucleoside analogues, such as gemcitabine, fludarabine, and cytarabine; the riboncleotide reductase inhibitor hydroxyurea; and the inhibitor of DNA polymerases alpha and gamma, aphidocolin. All interfere with the repair synthesis stage of various repair processes, including nucleotide excision repair. The potentiation of cisplatin cytotoxicity by treatment with aphidicolin has been studied extensively in human OC cell lines with variable synergism [63-65]. The likelihood of a significant improvement in the therapeutic index of cisplatin in refractory patients by the coadministration of a repair inhibitor is limited by the multifactorial nature typical of resistant tumor cells.

Platinum-DNA damage tolerance is a phenotype that has been observed in both cisplatin-resistant cells derived from chemotherapy-refractory patients and cells selected for primary cisplatin resistance in vitro. This phenotype may result from alterations in a variety of cellular pathways. One component of DNA damage tolerance observed in platinum-resistant cells involves loss of function of the DNA mismatch repair (MMR) system. The main function of this is to scan newly synthesized DNA and to remove mismatches that result from nucleotide incorporation errors made by the DNA polymerases [17]. In addition to causing genomic instability, it has been reported that loss of MMR is associated with low-level platinum resistance.

### Arsenic

Arsenic in its trivalent form is an interesting agent not only with inherent tumoricidal activity [66] but having multiple interactions that may enhance the cytotoxicity of cisplatin. In particular, arsenic may inhibit DNA repair [67], is clastogenic [68], induces stress response similar to heat shock [69], binds with glutathione and is exported by the multi-drug resistance protein MRP-1 [70], causes oxidative stress [71,72] and can induce apoptosis [73-78]. One cellular defense mechanism against cisplatin is dependent on glutathione conjugation and export by (MRP-1) [79]. Since arsenite is exported from the cell by the MRP-1 as a glutathione conjugate [70] it may compete for MRP-1 and reduce the efficiency of cisplatin export resulting in increased intracellular concentrations of cisplatin and the formation of more DNA adducts. Additionally, arsenite induces a stress response with substantial overlap with the heat shock induced stress response [69] with many of the same proteins being induced, including several heat shock proteins, heme-oxygenase and metallothionein.

Arsenic has a long history of usage as a medicinal. In western medicine, arsenic was used to treat chronic myelogenous leukemia until radiation treatment became commonplace in the 1930's [80]. Interest in arsenic as a chemotherapeutic was renewed when Chinese physicians reported success in treating acute promyelocytic leukemia (APL) with arsenic trioxide ("Pishi") also called "white arsenic" or "arsenolite". Another form "Xionghuang" is called "red arsenic" or "realgar" and realgar-containing traditional medicines are used in cancer treatments such as "Awei Huapi Gao" [81]. Arsenic trioxide (Trisenox^®^, As2O3) is now an FDA (Federal Drug Administration) approved chemotherapeutic for treating all-trans retinoic acid (ATRA) resistant APL [82]. There is much interest in the potential use of Trisenox^® ^to treat other malignancies (reviewed in [83,84]).

In vitro studies of arsenic trioxide induced cytotoxicity in human ovarian cancer cells are promising. Clinically achievable concentrations (2 μM) induced apoptosis in the platinum-resistant human ovarian cancer cell line CI80-13S and the platinum-sensitive human ovarian cancer cell line OVCAR. They also appeared to slow the growth of the cisplatin-sensitive human ovarian cancer cells GG and JAM [85]. Arsenic trioxide and cisplatin had additive effects on human ovarian carcinoma MDAH2774 cells [86]. Growth was slowed but apoptosis was apparently not induced in human ovarian carcinoma SKOV3 cells treated with arsenic trioxide in culture [87]. Arsenic trioxide induced apoptosis in human ovarian cancer 3AO cells and in a cisplatin-resistant derivative cell line 3AO/CDDP was associated with a large increase in percentage of cells expressing Fas [88]. These authors also reported biphasic dose-dependent alterations in cell cycle with increases in S-phase compartment associated with decrease in G2/M compartment at low (< 1 μM) arsenic trioxide and in G1 compartment at high (> 3 μM) arsenic trioxide concentrations. Arsenic trioxide decreased peritoneal metastasis of human ovarian cancer cells (3AO, SW626, HO-8910PM) injected intraperitoneally into nude mice, most likely because arsenic trioxide inhibited matrix metalloproteinase MMP-2 and MMP-9 expression and induced TIMP expression [89]. Thus, arsenic trioxide shows some promise as a single agent in treating EOC.

Arsenic trioxide may be useful in combination therapy. Polyunsaturated fatty acids appear to sensitize arsenic resistant tumor cells, including SKOV3 cells, to arsenic trioxide induced cytotoxicity and apoptosis [90]. There are two reports examining combined exposure to arsenite and cisplatin. One study with hepatocellular carcinoma cells suggests that arsenite and cisplatin act synergistically [91]. Another study reported that arsenite exhibited additivity with cisplatin, Adriamycin and etoposide in an ovarian and two prostate cancer cell lines [86].

The preliminary studies of arsenic trioxide discussed above suggest that arsenic trioxide may be useful in therapy for EOC particularly in combination chemotherapy. Consistent with this hypothesis is that preliminary studies in our laboratories suggest that arsenic trioxide in combination with hyperthermia can overcome cisplatin resistance in A2780/CP70 cells (manuscript in preparation). Clearly, further study is warranted.

## Conclusion

Despite modern standard therapy overall survival in women with ovarian cancer remains relatively poor. The most active chemotherapeutic agent remains cisplatin but ironically most patients whilst initially responding to cisplatin ultimately die with platinum-resistant disease. Arsenic is a promising agent for helping overcome platinum resistance. In addition to inherent tumoricidal activity it has multiple biochemical interactions that may enhance cisplatin cytotoxicity. Further research into this agent is needed.

## Competing interests

The authors declare that they have no competing interests.

## Authors' contributions

Both authors conceived the idea and jointly wrote the manuscript.

## About the author

C. William Helm is Associate Professor in the Division of Gynecologic Oncology at the University of Louisville and the James Graham Brown Cancer Center. His research interests include both normothermic and hyperthermic intraperitoneal chemotherapy (HIPEC) for ovarian cancer.

J. Christopher States is Professor of Pharmacology and Toxicology and Distinguished University Scholar at the University of Louisville. He is an established NIH investigator and recognized expert in disruption of mitosis by arsenicals.
